# Female sexual distress in infertile Turkish women

**DOI:** 10.4274/tjod.99997

**Published:** 2015-12-15

**Authors:** Serdar Aydın, Nihan Kurt, Selen Mandel, Mustafa Arda Kaplan, Nilay Karaca, Ramazan Dansuk

**Affiliations:** 1 Bezmialem Vakıf University Faculty of Medicine, Department of Obstetric and Gynecology, İstanbul, Turkey; 2 Bezmialem Vakıf University Faculty of Medicine, İstanbul, Turkey

**Keywords:** infertility, sexual distress, female sexual distress scale-revised, anti-Müllerian hormone, sexual dysfunction

## Abstract

**Objective:**

To evaluate the effect of infertility on sexual distress in women attending the infertility clinic.

**Materials and Methods:**

In a cross-sectional study we evaluated sexual distress among 88 women who attended the infertility clinic in our institute between January and June 2015. All women who were experiencing primary or secondary infertility during the study sampling were included in the sudy. Sexual distress was measured using the Female sexual distress scale-revised (FSDS-R), a cross-validated patient-reported outcomes measure. Correlations of FSDS-R with patient characteristics and laboratory measurements were calculated using Spearman’s rank correlation tests.

**Results:**

With the exceptions of the age of couples and serum anti-mullerian hormone (AMH) levels, no predictor of high sexual distress was found in the univariate analysis when comparing groups with regard to the FSDS-R cut-off score. The mean age of the sexually distressed women (33.6±5.8 years vs. 29.3±5.1 years) and their partners (35.4±4.8 years vs. 31.6±4.2 years) was significantly higher than those of the non distressed women, according to a FSDS-R score over 11 (p<0.05). The serum level of AMH was significantly lower in infertile women with high total sexual distress scores (1.4 vs. 7.6 ng/mL (p<0.001)).

**Conclusion:**

In infertile women, age of woman, age of partner, and serum AMH levels are related with the hope of women to have a child despite an association with sexual distress. Serum AMH, which is perceived as necessary for fertility, had a significant inverse correlation with levels of sexual stress.

## PRECIS:

We evaluated the effect of infertility on sexual distress in women requesting infertility treatment using the 13-item cross-validated Female sexual distress scale-revised.

## INTRODUCTION

Infertility is defined as failure of pregnancy in a married couple despite appropriately-timed intercourse. Impaired fertility has been estimated to affect 7-17% of all couples^(1)^. Marriage and having children are a learned purpose and a social responsibility for a couple^(2,3)^. Diagnosis of infertility causes a sudden and unexpected life crisis, and when the situation continues for an extended period it generates excessive stress and stretches the limits of adaptation mechanisms^(4)^. The majority of couples reported conflict, communication problems, and a differential investment in the process of infertility treatment. Inability to have a child or to become pregnant has unfavorable effects on marital relations, social life, emotional status, future plans, self-esteem, and the body image of women.

Infertility diagnosis, treatment process, and treatment outcome causes anxiety and fear in infertile couples^(5)^. After failure of artificial reproductive treatment, the prevalence of distress was reported to have increased from 33 to 43%; in some (8%), depression remained permanent. The duration of infertility has a high impact on the severity of depression^(6)^. Women reproach themselves and reflect anger and diminished self-confidence to their partners. This emotional response may cause conflict between the partners, develop feelings of inadequacy, and decrease self-esteem and the frequency of sexual intercourse.

Sexuality is an important issue that affects individuals and their social lives^(7)^. Sexual activity is not only for reproduction, sex is a way for men and women to become closer to each other and to satisfy physical, and psychological desires and needs. Sexual intercourse may become less spontaneous because it is performed only for reproduction and is restricted to days of increased fertility^(8)^. According to studies, infertile couples have some psychological disorders, including lack of marital satisfaction; impairment of relationships, especially between couples; lack of sexual satisfaction; loss of confidence in relation to sex and sexual intercourse; decreased libido; anger; and negative emotional effects^(9,10,11,12)^. These also reduce confidence in their fertility and affects their sexual life.

Relatively few studies have investigated the association of stress and infertility even though sexual dysfunction has been considered to be associated with infertility and many infertile couples experience stress in their sexual life^(13,14)^. Women with infertility often complain of problems in sexual function, but little is known about the specific nature of their stress and its level. Studies that assess sexual function and distress in female partners would be useful for counselling infertile couples, because normal sexual function is essential for human reproduction. Therefore, this study evaluated female sexual distress at the time of infertility treatment.

## MATERIAL AND METHODS

This cross-sectional study was undertaken at the infertility unit of the Bezmialem Vakıf University Faculty of Medicine, İstanbul, Turkey. The study was approved by the institutional ethics committee, which ensures ethical procedures in data collection and analysis. All women who were experiencing primary or secondary infertility during the study sampling were included. The treatments received include ovulation induction; ovarian stimulation and artificial insemination with husband’s spermatozoa; in vitro fertilization (IVF; and intra cytoplasmic sperm injection (ICSI). The inclusion criteria included married status; aged 18-45 years; lack of intense debate and controversy over the last month; and no stress events in the past three months. All women with physical and psychosocial problems, medical illness, addictions to alcohol or drugs and consumers of drugs that affect sexual function were excluded.

After the study protocol was explained and written informed consent was obtained, all eligible participants were asked to complete a self-administered questionnaire in a private room. One of the authors was available when participants needed further explanation about the questions. The first questionnaire was designed to gather information on demographic characteristics such as age, mode of delivery, education level, financial status, and smoking status. The second questionnaire was the Female sexual distress scale-revised (FSDS-R).

The FSDS-R is a patient-reported outcomes measure consisting of 13 items that assess different aspects of sexual activity-related distress in women^(15)^. Items are scored on a five-point Likert-type scale as 0=never, 1=rarely, 2=occasionally, 3=frequently, or 4=always. A total score, ranging from 0 to 52, can be computed by adding all 13 item scores together. Higher scores indicate higher levels of sexual distress. The original version of the FSDS-R demonstrated acceptable scale reliability with Cronbach’s alpha values ranging from α=0.87 to α=0.93 and high test-retest reliability (intra-class correlation coefficient ranging from r=0.74 to r=0.86)^(15)^. The FSDS-R was successfully cross-validated and a diagnostic cut-off score of >11 was shown to be highly effective in discriminating between women with hypoactive sexual desire disorder and other female sexual dysfunction (FSD), and those without FSD^(15,16)^.

Statistical analysis was performed after normality testing (histogram analysis and/or Kolmogorov-Smirnov) using IBM SPSS Statistics for Windows, Version 21.0. (Armonk, NY: IBM Corp.). Student’s t-test was used for comparisons of normally distributed variables, and the Mann-Whitney U test was used for categorical variables. Chi-square test and Fisher’s exact tests were used to compare the proportion of categorical variables. Correlations were calculated using Spearman’s rank correlation tests. Multivariable logistic regression models were developed to predict the probability of sexual distress using variables identified during univariate analysis. P<0.05 was considered statistically significant.

## RESULTS

Between January 2015 and June 2015, 92 (88.5%) of 100 women with infertility or the desire to have child fulfilled the inclusion criteria for the study. Four women refused to participate in the study because of the intimacy of the questions. The study included 88 infertile women of couples that sought medical care or an evaluation of couple infertility. None of the subjects in this study had co-morbid disease. No patient had a history of psychiatric disorders, sexual trauma or rape. Patients taking antidepressants or herbal drugs were not recruited in this study for potential effects on sexual function. The demographic and baseline characteristics of the participants are summarized in Table 1. The mean age was 30.1 years (range, 20-44 years), and the mean (SD) period of infertility in these couples was 2.9±1.1 years. The mean FSDS score of the women participating in the study was 5.2.

With the exceptions of age of both partners and serum anti-mullerian hormone (AMH) levels, no predictor of high sexual distress was found in the univariate analysis when comparing groups with regard to the FSDS-R cut-off score. Table 2 shows the comparison of the patients’ characteristics and laboratory measurements between infertile women with and without sexual distress. The mean age of infertile women (33.6±5.8 years vs. 29.3±5.1 years) and her partner (35.4±4.8 years vs. 31.6±4.2 years) were significantly higher in sexually distressed women compared with those without distress, according to a FSDS-R score over 11 (p<0.05). Mean Body mass indexes (BMI) were similar. No statistical difference between groups was found in the duration of infertility, education levels, and economic status. Percent of secondary infertility and causes of infertility were similar. Serum level of AMH was significantly lower in infertile women with high total sexual distress scores (1.4 vs. 7.6 ng/mL (p<0.001)).

Spearman’s correlation analysis showed a definite negative correlation between AMH level and FSDS-R scores (correlation coefficient-0.71; p≤0.001). Correlation of baseline characteristics, laboratory measures of patients, and FSDS-R scores are presented in Table 3. Age of the women, age of male partner, duration of infertility and marriage, BMI, number of previous unsuccessful IVF treatments, number of previous pregnancies, and level of FSH did not correlate with level of sexual distress in women.

Using variables identified during the univariate analysis, a multivariate logistic regression analysis was performed to assess whether a relationship existed between potential predictors that were detected in the univariate analysis. None of the other factors considered including age and partners’ age retained their significance. However, serum AMH level was the only identified variable that was independently associated with sexual distress (p=0.01, R^2^ 0.154).

## DISCUSSION

Lack of sexual satisfaction causes many psychological disturbances and marital discord. Therefore, in the present study we evaluated sexual distress of women with infertility. There are no data in the current literature about sexual distress of women with infertility. This is the first study showing the effect of infertility, treatment of infertility, and failure of treatment for sexual distress of women. Before the fifth edition of the Diagnostic and Statistical Manual (DSM-V) was released, the controversy about when sexual problems become sexual dysfunction and whether distress was required for FSD diagnosis persisted. Diagnostic categories of female sexual interest were based on the human sexual response model. However, recent reports put into question the validity of the model; both the strict distinction between different phases of arousal and the linear model of sexual response were found to inadequately explain sexual behavior, particularly in women^(17,18)^. This has in turn led to several proposed changes in sexual dysfunction diagnostic criteria^(19)^. Finally, DSM-V released the diagnosis category and according to the criteria, a woman has to report “significant distress” in order to be classified for a diagnosis of FSD^(20)^. Therefore, it is important to evaluate sexual distress for the diagnosis of sexual dysfunction in a common population.

Few studies have investigated the relationship between infertility and stress levels. The total infertility distress scale score, which was designed to measure infertility-related distress, was higher in women who did not work and those being treated for infertility for more than three years. The employment status of women, and physical, emotional, and sexual violence were reported to affect infertility-related general distress^(21)^. In another study, Ünal et al.^(22)^ reported that the infertility distress level decreased with increasing educational levels in women and increased with age. We found a significant difference in the age of women and their partners between women with and without high sexual distress. The education and socioeconomic levels were similar in our study. In contrast, no significant association relationship was found between age and education levels of women and the infertility distress level in another previous study^(21)^. Other authors found that problems with sexual function increased with age, but female sexual distress problems were more common in middle-aged women than in younger or older women^(23)^. This difference can be explained by the fact that general stress related with infertility was thought to arise from other factors such as a woman’s employment status, social environment, and violence. Other psychological variables such as coping and personality would also affect the sexual function. Women with strong tendencies toward introversion and emotional instability may be at a greater risk for sexual dysfunction^(24)^. Psychiatric evaluation of patients would lessen the bias caused by psychosocial variables.

In contrast to sexual distress, several studies have reported a decrease in sexual function in infertile women^(25,26)^. Infertility reduces sexual function in all domains but the significant decrease of sexual satisfaction found in infertile women is noteworthy^(27)^. In another study, sexual satisfaction and sexual function were examined in infertile women. The results of the study showed that infertile women had a significant decrease in all domains of function and sexual satisfaction^(27)^. Lee and Sun^(28)^ examined the psychosocial response of infertile couples and reported that physical and psychological effects of infertility were greater in infertile women compared with infertile men. The men experienced less stress and more confidence than women. In addition, infertile men had higher sexual satisfaction than their partners.

An increase in the duration of infertility is thought to cause an increase in stress by gradually decreasing women’s hopes of having children. In contrast to studies that reported that increased duration of infertility treatment increased the distress of infertile women, we failed to show significant differences in duration of infertility treatment between women with and without sexual distress^(21,22)^. The most interesting result of our study was a strong negative correlation with AMH and the female sexual distress score. AMH, a dimeric glycoprotein, is produced by granulose cells especially during the pre-antral and small antral follicle stages, independent of follicle-stimulating hormone^(29)^. Recent studies demonstrated that AMH was one of the most reliable predictors of the ovarian reserve and ovarian response to stimulation in assisted reproductive technologies (ART)^(30,31)^. The serum AMH concentration was found inversely correlated with increasing age, and thus serum concentrations of AMH decrease with advanced age accompanied by a concomitant decline in the number of primordial follicles^(32)^. AMH is also recognized as a good indicator for potential fertility. Therefore, AMH can affect expectations of couples and also physicians. We showed that primary or secondary infertility had no association with sexual distress. A study from Turkey evaluated the impact of the type of infertility on female sexual function and showed that women with secondary infertility had a higher prevalence of sexual dysfunction including sexual desire, orgasm, and satisfaction compared with primary infertile women^(33)^.

In the present study, we analyzed levels of sexual distress and factors that had potential to affect sexual distress but we did not evaluate the women’s sexual function or the partner’s sexual distress. Therefore, it can be considered as a limitation of the current study. The lack of use of the depression survey was another weak point of this study; however, but we excluded women with known depression and anxiety, and those who used antidepressants. We evaluated participants from a large section of the socio-economic spectrum; therefore, it can be said that the results of the research can be generalized to the entire research community. However, the study was conducted at a single center and the population of this study was composed of a specific group of Turkish women. This should also be noted as a limitation of this study; these data only reflect a group of Turkish women who received infertility treatment.

Taken together, these data support that infertility or treatment of infertility has specific psychological implications in the sex life of women. Age of women, age of partner, and serum AMH level was related with the hope of women to have a child and association with sexual distress of infertile women. Serum AMH, which is perceived as a predictor of the ovarian reserve or potential for fertility, had a significant inverse correlation with sexual stress levels. From our point of view, sexual counseling might help to overcome female sexual dysfunction in women with infertility. Further qualitative and quantitative research that investigate sexual distress and sexual dysfunction in infertile couples is needed.

## Figures and Tables

**Table 1:**
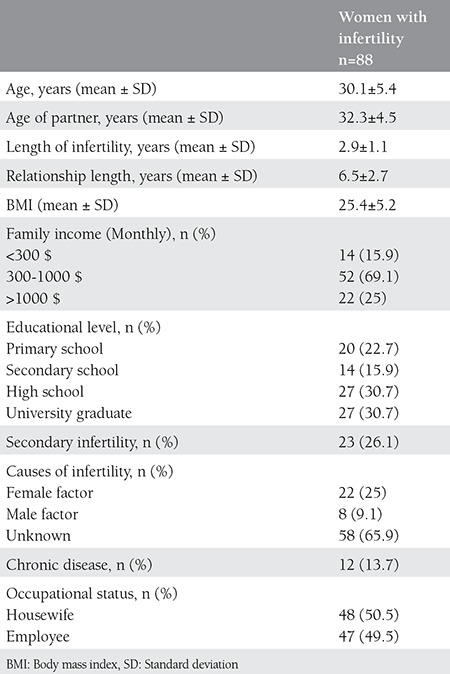
Demographic and baseline characteristics of participants with infertility

**Table 2:**
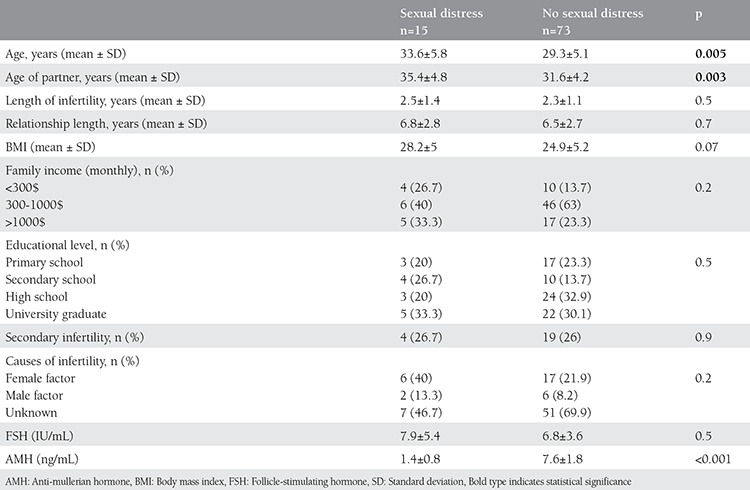
Comparison of patients’ characteristics and laboratory measurement between infertile women with and without sexual distress

**Table 3:**
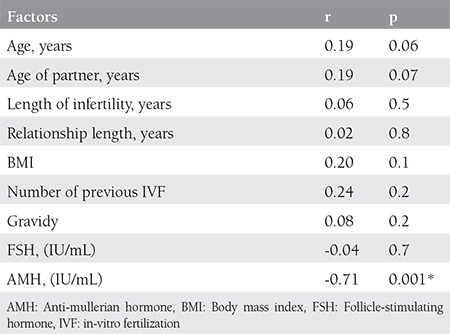
Correlations between patient baseline characteristics, laboratory measures and female sexual distress scale-revised score
